# Modulatory Effect of Polyphenolic Compounds from the Mangrove Tree *Rhizophora mangle* L. on Non-Alcoholic Fatty Liver Disease and Insulin Resistance in High-Fat Diet Obese Mice

**DOI:** 10.3390/molecules23092114

**Published:** 2018-08-22

**Authors:** Leonardo Mendes de Souza Mesquita, Cíntia Rabelo e Paiva Caria, Paola Souza Santos, Caio Cesar Ruy, Natalia da Silva Lima, Débora Kono Taketa Moreira, Claudia Quintino da Rocha, Daniella Carisa Murador, Veridiana Vera de Rosso, Alessandra Gambero, Wagner Vilegas

**Affiliations:** 1 UNESP—São Paulo State University/Coastal Campus of São Vicente, Laboratory of Bioprospection of Natural Products (LBPN) Pça Infante Dom Henrique S/N, 11330-900 São Vicente, SP, Brazil; mesquitalms@gmail.com (L.M.d.S.M.); claudiarocha3@yahoo.com.br (C.Q.d.R.); 2 Clinical Pharmacology and Gastroenterology Unit, USF—São Francisco University, Av. São Francisco de Assis, 218, 12916-900 Bragança Paulista, SP, Brazil; cintiarabello@yahoo.com.br (C.R.e.P.C.); pa.s.santos@hotmail.com (P.S.S.); ruyccaio@gmail.com (C.C.R.); lima.nat@gmail.com (N.d.S.L.); deboraktmoreira@gmail.com (D.K.T.M.); alessandra.gambero@usf.edu.br (A.G.); 3 Laboratório de Estudos Avançados em Fitomedicamentos (LEAF), UFMA—Federal University of Maranhão, Av. dos Portugueses, 1966-Bacanga, CEP: 65080-805 São Luís, Maranhão, Brazil; 4 Department of Biosciences, Federal University of São Paulo (UNIFESP), Silva Jardim Street, 136, Vila Mathias, 11015-020 Santos City, SP, Brazil; daniellamurador@gmail.com (D.C.M.); veriderosso@yahoo.com (V.V.d.R.)

**Keywords:** polyphenols, type 2 diabetes, non-alcoholic fatty liver disease, CD36, catechins

## Abstract

No scientific report proves the action of the phytochemicals from the mangrove tree *Rhizophora mangle* in the treatment of diabetes. The aim of this work is to evaluate the effects of the acetonic extract of *R. mangle* barks (AERM) on type 2 diabetes. The main chemical constituents of the extract were analyzed by high-performance liquid chromatography (HPLC) and flow injection analysis electrospray-iontrap mass spectrometry (FIA-ESI-IT-MS/MS). High-fat diet (HFD)-fed mice were used as model of type 2 diabetes associated with obesity. After 4 weeks of AERM 5 or 50 mg/kg/day orally, glucose homeostasis was evaluated by insulin tolerance test (kiTT). Hepatic steatosis, triglycerides and gene expression were also evaluated. AERM consists of catechin, quercetin and chlorogenic acids derivatives. These metabolites have nutritional importance, obese mice treated with AERM (50 mg/kg) presented improvements in insulin resistance resulting in hepatic steatosis reductions associated with a strong inhibition of hepatic mRNA levels of CD36. The beneficial effects of AERM in an obesity model could be associated with its inhibitory α-amylase activity detected in vitro. *Rhizophora mangle* partially reverses insulin resistance and hepatic steatosis associated with obesity, supporting previous claims in traditional knowledge.

## 1. Introduction

On the Brazilian coast, mangrove is one of the most representative biomes. This ecosystem is characterized as a transitional environment between terrestrial and aquatic ecosystems, and it is continuously threatened by real estate speculation and harbor construction [1]. Mangrove plants are potential sources of biologically active compounds and have wide application in ethnopharmacological practices. The habitat of these species is under stressful environmental conditions (salinity, temperature, tidal fluctuations, and anoxic soil). These plants are morphologically and physiologically adapted to this inhospitable environment. Therefore, they might present many substances which protect them from these adverse environmental [2].

Several medicinal plants are used as complementary therapy for diabetes, and studies demonstrating their efficacy and safety are still being developed [3]. Many reports document the increasing use of medicinal plants by modern populations in Mexico and Latin America [4]. No scientific report proves the action of the phytochemicals from the mangrove tree *Rhizophora mangle* in the treatment of diabetes. In this context, according to Andrade-Cetto and Heinrich, (2005), the barks of *R. mangle* L. (Rhizophoraceae) have been widely reported in traditional Mexican medicine to have antidiabetic property. Furthermore, *R. mangle* is commonly used in Latin America to treat inflammation, angina, asthma, pain, diarrhea, ulcers, tumors, and seizures [2,5,6].

Type 2 diabetes mellitus (T2D) is a major public health problem worldwide and the prevalence has been steadily increasing over the past few decades [7]. About 60% of Brazilian population is overweight or obese, and associated diseases such as T2D and non-alcoholic fatty liver disease (NAFLD) are becoming increasingly prevalent in the Brazilian population [8]. Diabetes is usually treated and managed using pharmacological drugs, which produce side effects, such as weight gain, edema, hypoglycemia, lactic acidosis, liver toxicity, and gastrointestinal disturbances after long-term use [9]. A recommended alternative for the treatment of diabetes is the use of traditional medicinal plants [10]. 

*Rhizophora mangle* L. (Rhizophoraceae) is one of the most prominent species in Brazilian mangrove ecosystems, which is a largely unexplored source of biological compounds with huge medicinal potential [11]. Phenolic compounds are the main source of phytochemicals found in mangrove plants, especially condensed tannins [12]; their barks are comprised of proanthocyanidins (PAs), oligomers, and polymers by flavan-3-ol, which have high antioxidant and anti-inflammatory activity [13]. PAs are widely distributed throughout the plant kingdom and are the second-most frequent phenolic substances, after lignins [14]. Polyphenols of plant origin can also help to prevent obesity and adipose tissue inflammation and improve obesity-associated metabolic syndrome in human subjects and animal models of obesity [15]. Our research group has previously obtained positive results with *R. mangle* acetone extract (AERM) for the treatment and prevention of gastrointestinal diseases [12,16]. However, a more detailed chemical analysis of AERM is needed to characterize the main chemical constituents.

Therefore, in this study, we characterized the chemical composition of the standardized AERM and investigated the effect of AERM on glucose homeostasis, adipose tissue inflammation, and hepatic steatosis in an experimental model of diet-induced obesity in mice. In addition, the potential antioxidant, anti-amylase, and anti-lipase of the extract was evaluated in vitro.

## 2. Results and Discussion

### 2.1. Chemical Characterization of Extracts

To obtain the most useful chemical information and optimal separation in the fingerprint chromatograms of AERM, the mobile phase compositions, gradient elution procedure, and detection wavelength were optimized. With the aim of enhancing resolution, glacial formic acid (FA) was added to the binary mixture of methanol–water. To acquire better selectivity and higher efficiency, different concentrations of FA (0.05%, 0.1% and 0.5%) in the aqueous phase were also investigated. The mobile phase consisting of a water-0.1% FA solution was chosen for the determination of AERM with many peaks on the chromatogram within 70 min. The wavelength was also optimized to obtain the highest number of compounds detected. 

**Figure 1:**
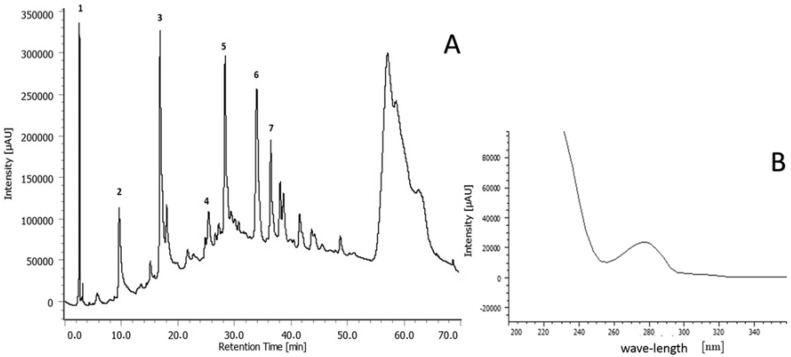
Compounds detected in the AERM (210 nm). Analytical HPLC-PDA chromatogram (**A**) and the corresponding UV spectra (peaks 1–7) (**B**).

Baseline resolution was optimized using the HPLC-PDA method. We observed seven main peaks in AERM, with maximum absorbance values around 232 and 278 nm, which covered more than 90% of the total chromatogram area (Figure 1A). Based on the UV spectrum of each chromatographic peaks (1–7) (Figure 1B), the constituents of AERM could be classified as catechin derivatives (λ_max_ 280 nm), which can polymerize, forming condensed tannins known PA [17]. The presence of condensed tannins in this extract was also confirmed by the presence of a low chromatographic resolution peak eluting between R_t_ 55–70 min. In spite of tannins are common in plants that occur in mangrove ecosystems [18], this work is the first to characterize and propose a fragmentation pathway of the condensed tannins in the acetonic extract from *R. mangle* barks. Oo et al. (2008) [19] and Zhang et al. (2010) [20] described the occurrence of catechins, epicatechins, and epigalocatechins in *R. apiculata* and *R. mangle*.

However, HPLC-PDA analyses were not sufficient to fully characterize the condensed tannins in AERM. According to Li et al. (2007) [21], ESI-MS techniques have been used efficiently for the characterization of several natural compounds, mainly polyphenolic compounds. Furthermore, MS/MS fragmentation of the flow injection analysis electrospray-ion trap mass spectrometry (FIA-ESI-IT-MS/MS) can generate product ions that give additional information about the structure of these compounds. FIA-ESI-IT-MS/MS has previously been applied to establish the polyphenol profile of complex matrices [22]. Thus, we decided to use this technique to obtain a qualitative metabolic fingerprint of the AERM, following clean-up using solid-phase extraction (SPE).

**Figure 2:**
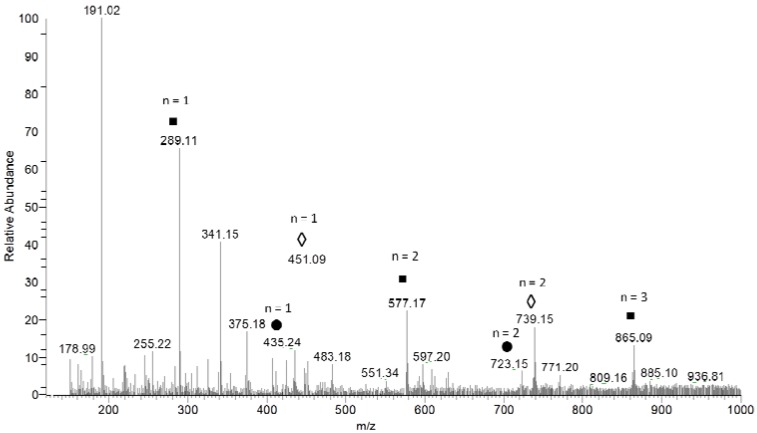
First-order mass spectrum of AERM evaluated by negative mode ionization. *n* = number of catechins units; ◾ catechin derivatives; ● deoxyhex-catechin derivatives; ◊ hexose-catechin derivatives.

To obtain qualitative information on PA in AERM, a sample rich in these compounds was prepared and directly injected into the ESI source of the mass spectrometer. We tested both positive and negative ionization, and the best results were obtained with negative mode. It has previously been reported that negative ionization is more sensitive and selective than positive ionization [23]. Figure 2 and Table 1 show the ESI-MS fingerprint obtained using full scan, showing the [M − H]^−^ ions of the compounds present in the extract. After MS/MS experiments with each peak observed in the full-scan spectrum, three main mechanisms of fragmentation were observed: retro-diels-alder (RDA), quinone methide (QM), and heterocyclic ring fission (HRF) [24]. The fragmentation patterns revealed the presence of three series of polymeric PAs. In the full-scan experiment, the *m*/*z* 289 ion represented one unit of catechin. In addition, we observed a first series of ions separated by 288 Da corresponding to ion peaks of dimeric (*m*/*z* 577) and trimeric (*m*/*z* 865) PA. We detected a second series of PAs, based on a catechin core linked to hexose moieties (*m*/*z* 451: monomer; *m*/*z* 739: dimer). A third series of PAs is represented by catechins linked to deoxyhexose moieties (*m*/*z* 435: monomer; *m*/*z* 723: dimer).

**Figure 3:**
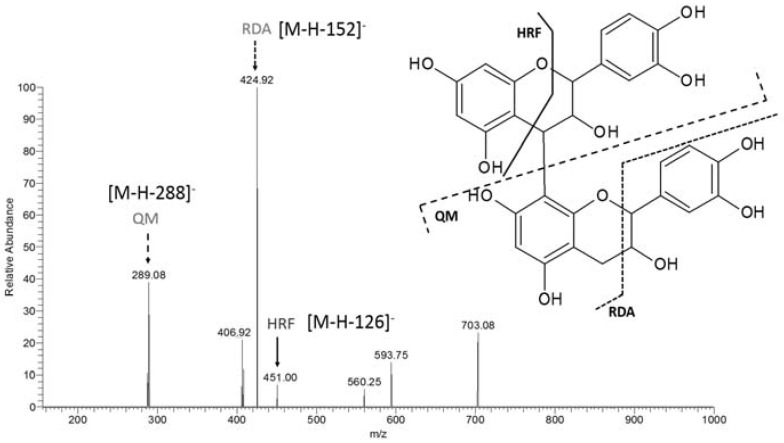
MS/MS spectrum of the ion at *m*/*z* 577 evidencing the main patterns of fragmentation of PAs. HRF = heterocyclic ring fission; RDA = Retro Dies-Alder fragmentation; QM = quinone-methide fragmentation.

The MS² spectrum of the ion at *m*/*z* 577 (Figure 3) showed major fragments at *m*/*z* 451, 425, and 289. The ion at *m*/*z* 451 arises from the loss of 126 mass units, corresponding to the HRF fragmentation. The ion at *m*/*z* 425 was derived from the loss of 152 Da from the precursor ion at *m*/*z* 577 and was proposed to arise from an RDA fragmentation. The fragment at *m*/*z* 289 [M-288-H]^−^ was assigned to a QM fragmentation of the catechin dimer. These fragmentation patterns have been previously described and confirm the presence of PAs [25].

**Figure 4:**
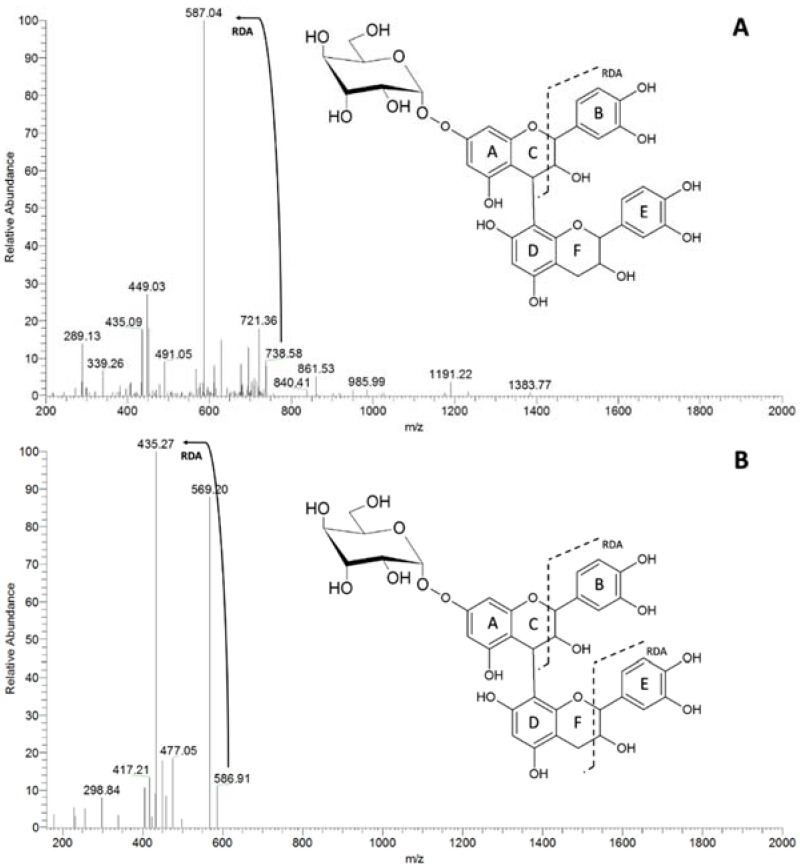
MS/MS spectra of the ion at *m*/*z* 739, evidencing the main patterns of fragmentation of hexosyl PAs. RDA = retro Dies-Alder. (**A**) MS² spectrum of the precursor ion of *m*/*z* 739 [M-152-H]^−^; (**B**) MS³ spectrum of the precursor ion of *m*/*z* 739 [M-152-152-H]^−^.

The PA series containing hexose-catechins was also investigated using the same approach. The MS² spectrum of the ion at *m*/*z* 739 generated the product ion at *m*/*z* 587 [M-152-H]^−^, due to RDA fragmentation (Figure 4A), followed by another RDA [M-152-152-H]^−^ fragmentation, generating the ion at *m*/*z* 435 (Figure 4B). This fragmentation pattern allowed us to presume the position of the sugar moiety either at ring A or D (Figure 4A,B). Similar fragmentation patterns occurred with the third PA series containing a deoxyhexose moiety (*m*/*z* 435 and 723).

**Figure 5:**
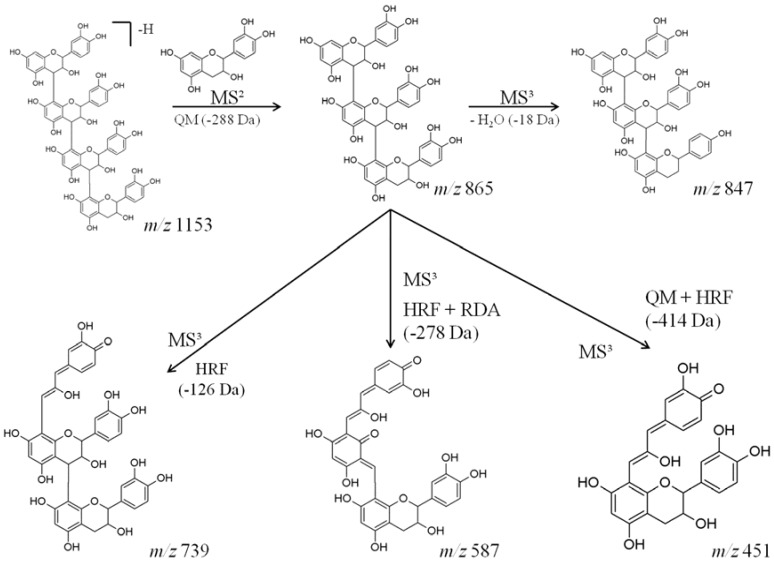
Fragmentation pathways of a possible tetramer proanthocyanidin found in AERM. The main fragmentation mechanisms involved are: HRF, RDA, and QM.

However, we found that this type of fragmentation pattern does not occur with molecules of higher molecular weight, such as catechin tetramers (*m*/*z* 1153). The MS² spectrum of the precursor ion at *m*/*z* 1153 generated the product ion at *m*/*z* 865 [M-288-H]^−^, due to QM fragmentation (Figure 5). The MS³ spectrum of the precursor ion at *m*/*z* 1153 [M-288-H]^−^ showed major product ions at *m*/*z* 847, *m*/*z* 739, *m*/*z* 587 and *m*/*z* 451 (Figure 5). The product ion at *m*/*z* 847 was derived from the loss of water [M-18-H]^−^. The product ion at *m*/*z* 739 was due to HRF [M-126-H]^−^. HRF + RDA yielded the product ion at *m*/*z* 587 [M-278-H]^−^; and the product ion at *m*/*z* 451 was generated from a QM + HRF fragmentation [M-414-H]^−^. Figure 5 shows the fragmentation pathway proposed for the higher molecular weight molecules, which possessed mixed fragmentations that were not detected in smaller molecules.

**Figure 6:**
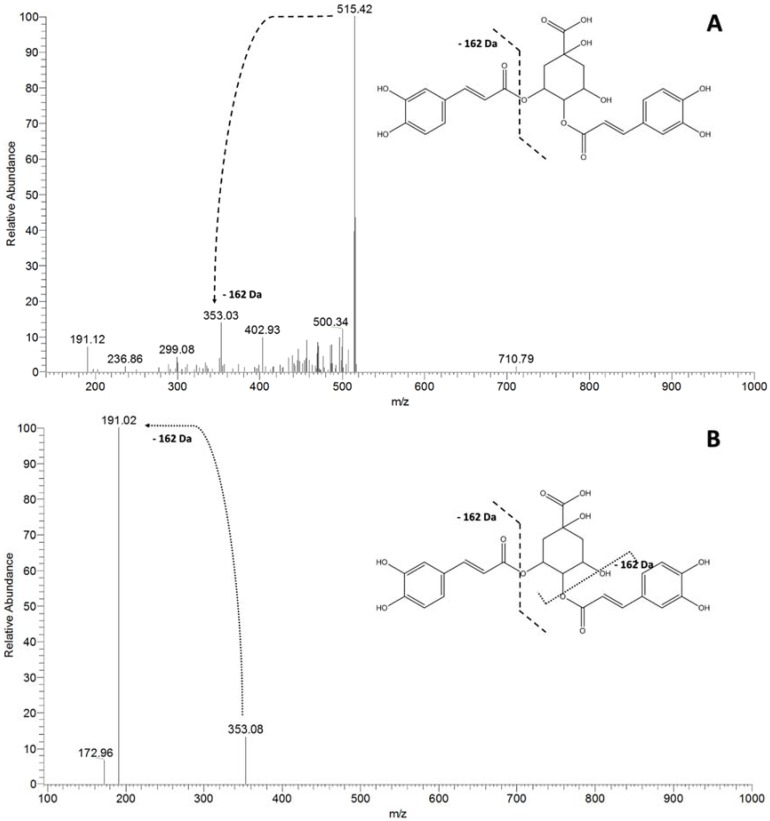
MS/MS spectrum of the precursor ion at *m*/*z* 515, evidencing the main patterns of fragmentation of dicaffeoyl-quinic acids. (**A**) The MS² spectrum of the precursor ion at 515 [M-162-H]^−^; (**B**) The MS³ spectrum of the precursor ion at *m*/*z* 515 [M-162-162-H]^−^.

Although the acetone extract of *R. mangle* was composed mostly of catechin derivatives, other substances were detected. Quinic acid derivatives (*m*/*z* 191) were detected in the negative mode. MS^2^ fragmentation of the precursor ion at *m*/*z* 515 generated the product ion at *m*/*z* 353 [M-162-H]^−^ (Figure 6A, Table 1). The MS³ fragmentation of the precursor ion of *m*/*z* 515 produced the product ion of *m*/*z* 191 [M-162-162-H]^−^ (Figure 6B, Table 1), which is characteristic of dicaffeoyl-quinic acids [26]. It is probable that these substances are produced owing to the biotic and abiotic stress conditions to which the plant is exposed in the ecosystem [26]. In addition, compounds in this class possess antioxidant, antiviral, anti-bactericidal, and anti-inflammatory effects, have been shown to reduce the risk of cardiovascular disease and type 2 diabetes, and have a beneficial role in Alzheimer’s Disease [27]. To our knowledge, this class of substances has not been previously reported in the literature for the genus *Rhizophora*.

**Figure 7:**
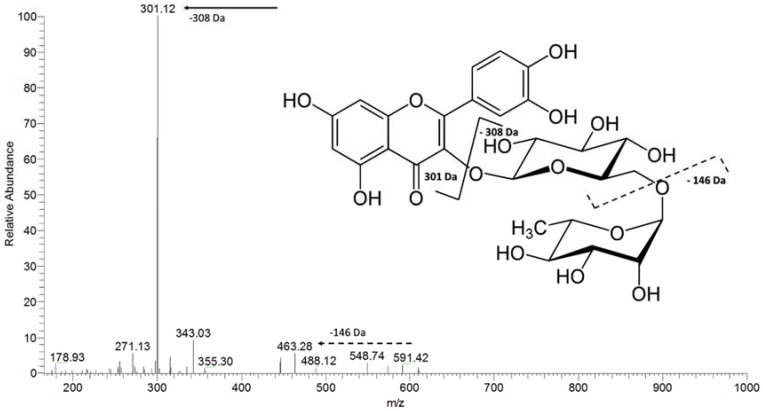
The MS² spectrum of the ion at *m*/*z* 609, evidencing the main patterns of fragmentation of rutin.

Another substance detected by FIA-ESI-IT-MS presented *m*/*z* 609. The MS² fragmentation of the precursor ion *m*/*z* 609 produced two major fragments. The ion at *m*/*z* 463 [M-146-H]^−^ (Figure 7) was probably due to the loss of a deoxyhexose moiety, whereas the ion at *m*/*z* 301 [M-308-H]^−^ (Figure 7) arose from the loss of a glycosidic hexose-deoxyhexose chain, which led us to propose the presence of rutin, a flavonoid commonly found in several plant families that has previously been detected in *R. mangle* extracts [28].

### 2.2. In Vitro Activities of the Extract

The relatively stable organic radical 2,2′-azino-bis(3-ethylbenzothiazoline-6-sulphonic acid) (ABTS^●+^) has been widely used to determine the radical scavenging activity of different plant extracts [29]. The AERM demonstrated intense antioxidant activity (608.8 μmol Trolox/g). Zhang et al. (2010) [20] reported the powerful antioxidant activity of *R. mangle* and *R. mucronata* from the leaf ethanolic extracts using the, 2-diphenyl-1-picrylhydrazyl assay (DPPH) and attributed this effect to the large amount of condensed tannins present in the extract. Takara et al. (2008) [30] evaluated the antioxidant activity of the *R. stylosa* species and showed that the sugar moieties present in the condensed tannins further increase the efficiency of free-radical sequestration, which might occur in AERM.

**Figure 8:**
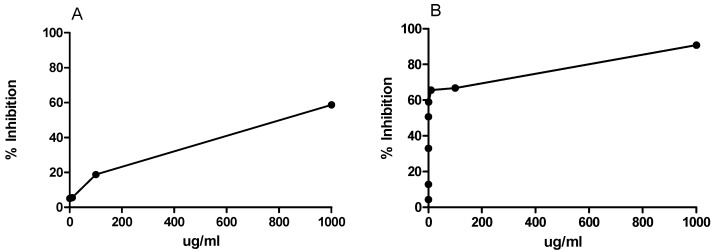
In vitro pancreatic lipase (**A**) and amylase (**B**) inhibition by AERM. Experiment was performed in triplicate. IC_50_ was calculated from curves using GraphPad Prism Software.

The digestion of food starch and triglycerides in the gastrointestinal milieu is performed by amylases and lipases, respectively. Acarbose is a potent inhibitor of amylases, and delays the production of glucose helping to improve insulin resistance and glucose homeostasis in diabetic patients [31]. Similarly, orlistat, a pancreatic lipase inhibitor currently approved as an anti-obesity therapeutic, works by reducing the intestinal absorption of free fatty acids [32]. The presence of lipase and/or amylase inhibitors of plant origin has been demonstrated for different polyphenolic-rich plant species [33]. AERM inhibited lipase activity with an inhibitory concentration IC_50_ of 803 µg/mL, while orlistat inhibits lipase with an IC_50_ of 27.4 µg/mL in the same set of assays. Alpha-amylase assay resulted in the inhibition of AERM activity at an IC_50_ of 19.0 µg/mL, while acarbose inhibited amylase with an IC_50_ of 5.2 µg/mL (Figure 8). Tannins have been suggested as potent inhibitors of human salivary and porcine pancreatic α-amylases [34,35], suggesting that PAs from AERM could be implicated in this potent inhibitory activity. In addition, it has been reported that PAs of cinnamon water extract inhibited the amyloid formation of Human islet amyloid polypeptide (hIAPP) in a dose-dependent manner. Proanthocyanidins affected the secondary structures of hIAPP and delayed the structural transition from unstructured coils to β-sheet-rich structures. The PAs are the major components of the AERM, the causes for the antidiabetic effect of the extract are considered [36].

### 2.3. Hepatoprotective and Insulin Sensitization after AERM Administration In Vivo

Mice fed a high-fat diet (HFD) for 8 weeks became obese compared with mice fed a standard diet, as observed by the increase in final body weight and visceral and subcutaneous adipose tissue deposits (Table 2). An increase in liver weight was also observed in the obese mice, suggesting the presence of hepatic alterations associated with obesity (Table 2). Four-weeks treatment with AERM was not enough to significantly reduce the body weight gain and adiposity in HFD and control mice; however, liver weight was significantly reduced in obese mice treated with *R. mangle* compared with non-treated obese mice (Table 2).

**Figure 9:**
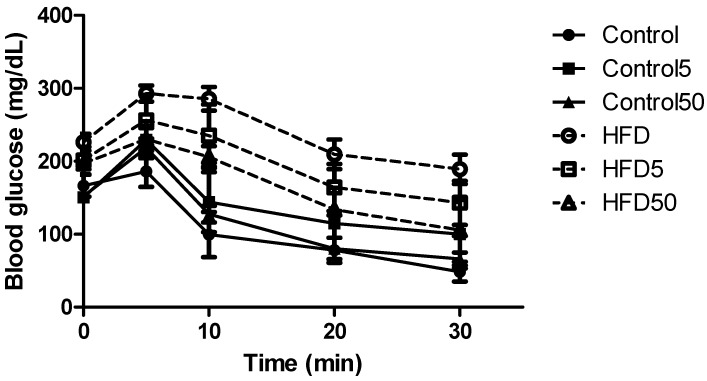
Blood glucose before (0) and after insulin administration in control mice, control mice treated with AERM 5 mg·kg^−1^ (Control5) or control mice treated with AERM 50 mg·kg^−1^ (Control50), obese mice (HFD), obese mice treated with AERM 5 mg·kg^−1^ (HFD5) or obese mice treated with AERM 50 mg·kg^−1^ (HFD50). The results are expressed as the means ± standard errors of the mean (*n* = 5).

Interestingly, fasting blood levels of glucose and insulin were also reduced in obese mice treated with higher-dose AERM at (50 mg/kg), and these mice were partially more tolerant to insulin, as observed by the k of insulin tolerance test (ITT) value and ITT curves (Table 3 and Figure 9). Serum total and low-density lipoprotein (LDL)-cholesterol were also reduced by AERM. Insulin resistance that results in hyperglycemia/hyperinsulinemia is routinely associated with obesity. Oral quercetin supplementation (30 mg/kg/day) was not effective at inducing weight loss in HFD mice, but it was effective as an antidiabetic [37]. As demonstrated, water provided orally ad libitum with 0.5% of procyanidins from apple juice over 4 weeks to genetically obese mice resulted in the maintenance of body weight and adiposity. It also improved insulin resistance via the suppression of pro-inflammatory cytokines in the liver [38].

**Figure 10:**
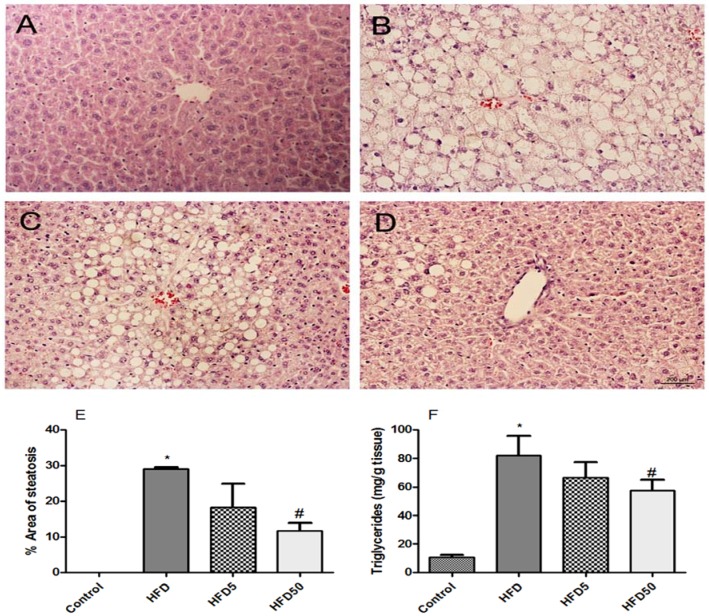
(**A**) Liver histology of control mice; (**B**) obese mice (HFD); (**C**) obese mice treated with AERM 5 mg·kg^−1^ (HFD5); (**D**) obese mice treated with AERM 50 mg·kg^−1^ (HFD50); (**E**) steatosis measurement; (**F**) triglycerides content. Hematoxylin–eosin staining of 5.0 μm liver sections. Magnification: 200×. Steatosis measurement in five random power fields of five mice per group. (**E**–**F**) The results are expressed as the means ± standard errors of the mean (*n* = 5). * *p* < 0.01 when compared with the control group and ^#^ *p* < 0.05 when compared with the HFD group.

**Figure 11:**
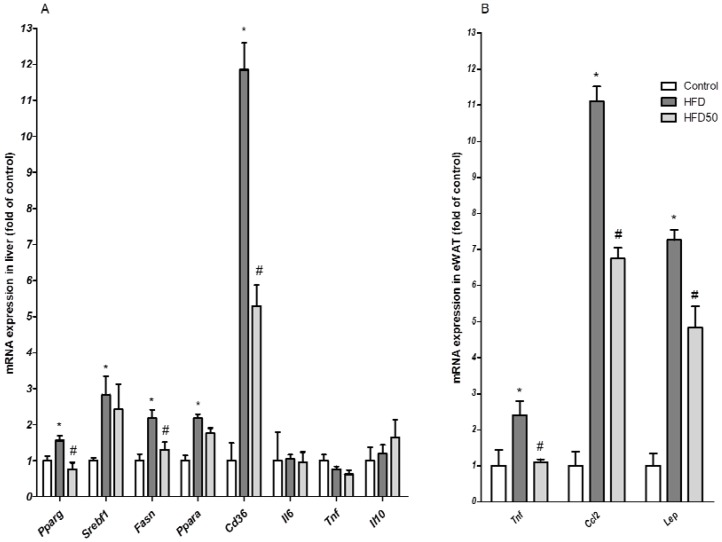
(**A**) mRNA expression in the liver and (**B**) adipose tissue of control mice (Control), obese mice (HFD) and obese mice treated with AERM 50 mg·kg^−1^ (HFD50). The results are expressed as the means ± standard errors of the mean (*n* = 5). * *p* < 0.05 when compared with control group and ^#^ *p* < 0.05 compared with the HFD group.

The analysis of body composition, as described above, revealed that obese mice treated with both AERM doses had reduced liver weight compared with non-treated obese mice (Table 2). Obese mice presented steatosis after receiving an HFD for 12 weeks; however, in mice treated with AERM at 50 mg/kg, the area of hepatic steatosis and the triglycerides content were significantly reduced (Figure 10). Recently, it has been reported that catechins can improve the blood lipid profile, and to prevent the accumulation of fat in the liver on hyperlipidemic rats [39]. However, in Swiss mice fed an HFD for 12 weeks, no increase in the expression of hepatic pro-inflammatory cytokines (Interleukin (IL)-6, Tumor Necrosis Factor (TNF)) were detected (Figure 11). Feeding C57Bl6 mice an HFD for 12-weeks was unable to induce inflammatory markers or c-Jun N-terminal kinases (JNK) activation in liver. Hepatic inflammation was only observed when fructose or sucrose was added to the HFD [40]. Accumulation of hepatic triglycerides (steatosis) is the first step of NAFLD, and may be due the increased free fatty acid (FFA) supply, decreased FFA oxidation, increased de novo lipogenesis, and/or very low-density lipoprotein (VLDL)-triglyceride secretion [41]. Liver uptake of FFA is facilitated by cell-surface receptors, such as CD36/fatty acid translocase. The mRNA expression of CD36 in hepatocytes is normally low, but an important increase was observed in response to a HFD and following the activation of nuclear receptors, including peroxisome proliferator-activated receptor (PPAR)-γ [42]. We observed a modulatory effect of AERM on the mRNA expression of liver PPAR-γ, which was associated with the inhibition of CD36 mRNA expression (Figure 11), suggesting that AERM down regulates CD36 mRNA via PPAR-γ inhibition. Hepatic expression of *Srebf1* and *Pparα*, which encode sterol regulatory-binding protein-1c (SREBP-1c), a regulator of de novo lipogenesis, or PPAR-α, a regulator of FA oxidation, was not altered following AERM treatment. Herbal formulations containing the aqueous extract of *Dolichos lablab*, the aqueous extracts of *Penthorum chinense*, the ethanol extract of *Solidago virgaurea*, or the isolated flavonoid quercetin, could able to inhibit hepatic lipid accumulation through the downregulation of mRNA expression of CD36 [43,44,45,46]. Several studies have demonstrated that orlistat in the range 10–100 mg/kg can reduce liver weight, hepatic triglycerides, and serum triglycerides/cholesterol in mice fed a HFD [47,48,49]. AERM demonstrated in vitro anti-lipase activity, which was less potent than that of orlistat, but in vitro anti-amylase was more similar inhibited if compared to acarbose. In vivo administration of orlistat requires doses around 50 mg/kg to determine lowering serum triglycerides (TG) effects and probably it explain why we did not observe it in vivo with AERM. However, in vivo, acarbose is only effective at 40 mg/kg/day [50,51], suggesting that anti-amylase activity could be a role for glucose homeostasis and for the hepatoprotective effects of AERM. 

Interestingly, mice treated with AERM presented reduced expression of pro-inflammatory genes but no reduction in adiposity compared with non-treated obese mice (Figure 6B), suggesting that AERM has anti-inflammatory activity, mitigating obesity-associated adipose tissue inflammation, which could contribute to reverse insulin resistance. A similar effect as previously described for dietary supplementation with grape-seed PAs [52] and isolated catechins [53]. However, we investigated the antioxidant effects of AERM by measuring malondialdehyde (MDA) as an index of lipid peroxidation [54] in the liver and adipose tissue of mice, and were unable to confirm that the antioxidant activity was maintained in vivo (data not shown).

Food ingestion was not different between treated and non-treated obese groups. However, the higher dose of AERM employed in our study was able to reduce the amount of food intake by the control group, as well as increase the basal insulin level (Table 2). Insulin can signal energy homeostasis to the central nervous system. Insulin and leptin levels are increased in individuals with obesity, but due to insulin resistance in these individuals, the satiety signal is dysregulated [55,56]. AERM-induced appetite suppression was only observed in lean mice, without insulin resistance, showing that the central anorexigenic action of this protein is being performed correctly; however, the mechanism(s) through which AERM increases insulin release should be further investigated. Oral administration of a tetrameric procyanidin from cacao, cinnamtannin A2, in lean mice resulted in incretin activity by increasing insulin release through mechanisms involving increased plasma levels of glucagon-like peptide (GLP)-1 [57].

## 3. Materials and Methods

### 3.1. Sample Taxon

The barks of *Rhizophora mangle* L. (Rhizophoraceae) were collected from the estuarine system of the ecological station at Juréia-Itatins (Peruíbe, São Paulo—Brazil—24°25′40″ S–47°05′20″ W). Collection of the material was approved by prior authorization from the Brazilian authorities (IBAMA/MMA: 52497-1). A voucher specimen (no. 11459) has been deposited at the Herbarium HUSC of the Santa Cecilia University (Santos, São Paulo—Brazil).

### 3.2. Chemical Characterization

#### 3.2.1. Preparation of Plant Extract

Fresh barks of *R. mangle* were washed, shade-dried, powdered in a knife mill, and sieved through a #60 mesh sieve. The powder (50 g) was extracted with 0.5 L of acetone (70% *v*/*v*) and macerated for 7 days at room temperature (24 °C), protected from light. The macerate was filtered through Whatman no.1 filter paper and concentrated in a rotary flash evaporator at a temperature not exceeding 35 °C. The extract (7 g, 14%) was lyophilized and stored in amber bottles and placed into in a freezer (−40 °C). To minimize the interference of very high-order polymeric compounds, a SPE was performed. An aliquot (10 mg) of the extract was submitted to the SPE using a RP18 cartridge, eluted with H_2_O/MeOH 8:2 (*v*/*v*) (5 mL). The eluate was filtered through the nylon membrane (0.22 µm) and directly analyzed by chromatography and mass spectrometry.

#### 3.2.2. HPLC-PDA

The chemical composition of AERM was investigated by high-performance liquid chromatography coupled to a Photodiode Array Detector (HPLC-PDA), using a Jasco (Tokyo, Japan) HPLC equipped with a PU-2089 quaternary solvent pump, a MD-2010 PAD, and an AS-2055 autosampler. The analytical column, maintained at room temperature (25 °C), was a Phenomenex Synergi Hydro RP18 (250 mm × 4.6 mm H × i.d.; 4 µm) with a Phenomenex security guard column (4.0 × 2.0 mm). Phenolic acids, flavonoids, flavan-3-ols, and PA were separated using the mobile phase of water (eluent A) and acetonitrile (eluent B), solvent A containing 0.1% formic acid, with the following gradient program: 5–50% B (30 min), 30–85% B (30–35 min), isocratic 85% B (45 min), 85–100% B (45–70 min), return to 5% B (2 min), and the column was re-equilibrated under the initial conditions for 18 min before the next injection. The flow rate was 1.0 mL·min^−1^, and the total run time was 70 min. EZChrom Elite Data System software (Chromatec, Idstein, Germany) was used for detection operation and data processing. Compounds were identified by comparing retention times and UV spectral analyses.

#### 3.2.3. FIA-ESI-IT-MS/MS

Flow injection analysis (FIA) was performed using a Thermo Fisher Scientific ion trap mass spectrometer (San Jose, CA, USA) equipped with an electrospray ionization source. MS and MS/MS analyses in negative ion mode were selected after calibration infusing a standard solution of (+)-catechin (1 µg/mL in methanol) at a flow rate of 5 µL/min under the following conditions: capillary voltage −31 V, spray voltage 5 kV, tube lens offset 75 V, capillary temperature 300 °C, sheath gas (N_2_) flow rate 8 (arbitrary units). Negative ion mass spectra were recorded in the range *m*/*z* 100–2000 Da. The first event was a full-scan mass spectrum to acquire data on ions in the *m*/*z* range. The second scan event was an MS/MS experiment performed using a data-dependent scan carried out on deprotonated molecules from the compounds at collision energy of 25–30% and activation time of 30 ms. Data were acquired and processed using the Xcalibur software (version 2.2 SP1.48).

### 3.3. In Vitro Assays

#### 3.3.1. Trolox Equivalent Antioxidant Capacity (TEAC) Assay

2,2′-azino-bis(3-ethylbenzothiazoline-6-sulphonic acid) (ABTS) and potassium persulfate were dissolved in distilled water to a final concentration of 7 and 2.45 mM respectively. These two solutions were mixed, and the mixture was allowed to stand in the dark at room temperature (24 °C) for 16 h before use, to allow the production of ABTS radical (ABTS•^+^). To study phenolic compounds, the ABTS radical solution was diluted with distilled water to obtain an absorbance of 1.00 at 734 nm. AERM (final concentrations 0.0001–0.01 mg/mL) or Trolox standards (final concentration 0–20 mM) were added to diluted ABTS•^+^ solution and the absorbance reading was taken 6 min after mixing, using a spectrophotometer. Results are presented as the ability of phenols to scavenge free-radical ABTS•^+^ (Trolox equivalent antioxidant capacity).

#### 3.3.2. Lipase Activity Assay

Lipase activity was determined by measuring the release of p-nitrophenol from p-nitrophenyl palmitate (4-NPP) via a spectrophotometric method at 405 nm. Lipase (10 mg/mL) from porcine pancreas type II (Sigma, St. Louis, MO, USA) was dissolved in reaction buffer 50 mM Tris–HCl pH 8.0, and then centrifuged at 5000 *g* for 5 min to remove the insoluble components. 4-NPP was dissolved in 1:9 *v*/*v* isopropanol:reaction buffer (50 mM Tris–HCl pH 8,0 containing 0.1% Arabic gum and 0.4% de triton x-100). The final reaction mixture was 1:2:1 *v*/*v* AERM:pancreatic lipase solution:4-NPP (10 mM) and was added into wells of a 96-well microplate and incubated at 37 °C for 20 min, after which the amount of 4-nitrophenol released was measured. Orlistat was used as a positive control. Each experiment was performed in triplicate. Lipase inhibition was expressed as percentage and the IC_50_ was calculated by using GraphPad Prism Software (version 5.01).

#### 3.3.3. Alpha-Amylase Activity Assay

α-amylase activity was quantified by the reduction of 3,5-dinitrosalicylic acid to 3-amino-5-nitrosalicylic acid. α-amylase (10 mg/mL) from porcine pancreas type VI-B (Sigma St. Louis, MO, USA) was dissolved in 50 mM Tris–HCl buffer, pH 7.0, containing NaCl 38 mmol/L and CaCl_2_ 0.1 mmol/L. The reaction included 0.1% potato starch (*w*/*v*) as substrate, incubated at 37 °C for 20 min with AERM and α-amylase solution. The reaction was interrupted with the addition of 3,5-dinitrosalicylic acid (DNS), heated at 100 °C for 5 min, prior to quantification by spectrophotometry at 540 nm. Activity was calculated using a glucose standard curve as a reference. Acarbose was used as a positive control. The experiment was carried out in triplicate. α-amylase inhibition was expressed as percentage of inhibition and the IC_50_ was calculated using GraphPad Prism Software (version 5.01).

### 3.4. Experimental Model of Diet-Induced Obesity

#### 3.4.1. Animals

Swiss male mice, specific-pathogen free, were obtained from the Multidisciplinary Center for Biological Research (CEMIB; State University of Campinas, Campinas, SP, Brazil). Experiments were performed in accordance with the principles outlined by the National Council for the Control of Animal Experimentation (CONCEA, Brazilia, Brazil) and received approval from the Ethics Committee of São Francisco University, Bragança Paulista, SP, Brazil (Protocol 001.02.16). Animals were maintained on a 12:12 h artificial light–dark cycle under controlled humidity and temperature. 

#### 3.4.2. Diet-Induced Obesity and *R. mangle* Treatment

Mice (six-week-old) were maintained on commercial chow (Control; 5% energy from fat) or HFD (60% energy from fat), as previously described [56]. Body weight was assessed weekly. After 8 weeks, the control and HFD animals were randomly divided into groups (*n* = 5 each). During the next 4 weeks, mice received 5 or 50 mg/kg daily of AERM by oral gavage, dissolved in filtered tap water adjusted in a final volume of 4 mL/kg. Untreated groups received only water by gavage. Food intake was monitored by subtracting the amount of food consumed from the volume offered to the animals during AERM treatment.

#### 3.4.3. Blood Glucose Levels and Insulin Tolerance Tests

The insulin tolerance test was performed twenty-four hours before the end of the protocol [58], in which after 6 h of fasting, blood samples were collected from the tails of the mice to measure basal blood glucose using a glucometer (Accutrend Plus, Roche Diagnostics, Mannheim, Germany). Insulin (1.5 U/kg) was administered intraperitoneally and glucose blood was measured after 5, 10, 20 and 30 min. Results were calculated using the formula 0.693/t1/2 and expressed as kITT, which the glucose t1/2 was calculated from the slope of the least-square analysis during the linear decay phase.

#### 3.4.4. Necropsy and Sample Collection

Mice were fasted overnight and anesthetized by xylazine/ketamine overdose (0.1 mL/30 g body weight of 1:1 *v*/*v* of 2% xylazine and 10% ketamine). Blood was collected by cardiac puncture. Adipose tissue deposits (epididymis and subcutaneous), liver, and gastrocnemius muscle were carefully dissected, weighed, and expressed as a percentage of body weight (b.w.). Liver and visceral adipose samples were collected and stored at −80 °C for further analyses.

#### 3.4.5. Hepatic Analyses

Hydrated 5.0 μm sections of paraformaldehyde-fixed, paraffin-embedded liver specimens were stained using hematoxylin and eosin stain to evaluate the presence of liver steatosis. Steatosis was quantified by counting steatosis (macrovesicular and microvesicular) against a grid of 144 points. For the total lipid extractions, liver samples were homogenized in NaCl 0.9%, and then a chloroform and methanol mixture (2:1 *v*/*v*) was added [59]. The chloroform layer was collected, dried under N_2_, and reconstituted in PBS buffer. Triglycerides and total cholesterol were measured using a commercial kit (LaborLab, MG, Brazil).

#### 3.4.6. Serum Analyses

Serum triglycerides and total LDL and high-density lipoprotein (HDL) cholesterol were measured using commercial kits (LaborLab, MG, Brazil). Insulin was quantified using a Milliplex kit (Merck Millipore, Burlington, MA, USA) [60].

#### 3.4.7. Quantitative Real-Time Polymerase Chain Reaction (qPCR)

The relative expression of genes in the liver and adipose tissue samples were quantified by real-time polymerase chain reaction (PCR) as described previously [58]. All data (analyzed by the ΔΔCt method) were normalized to a control gene (18S) and represented as fold change with respect to the Control group. Primers used are listed in Table 4.

### 3.5. Statistical Analyses

The results were expressed as the means ± standard errors of the mean. Statistically significant differences were determined using analysis of variance (ANOVA) followed by Dunnett’s test for multiple comparisons, using GraphPad InStat (GraphPad Software, Inc., La Jolla, CA, USA). *p* Values < 0.05 were considered significant.

## 4. Conclusions

In summary, these data provide evidence for the presence of three PA series, from one to four catechin moieties and their glycosylated forms, as well as phenolic acids and flavonoids, in the acetonic extract of *R. mangle* barks, which displayed anti-lipase, and anti-amylase activity in vitro. These compounds displayed an intense effect on the lipid metabolism and therefore help in the comorbidities associated with diabetes and obesity. in vivo, *R. mangle* bark extracts displayed hepatoprotective effects associated with the improvement of insulin resistance and potential effects upon adipose tissue inflammation associated with obesity. Taken together, these results support traditional knowledge of the use of *R. mangle* for the treatment of type 2 diabetes and reveal that bark extract has potential for the treatment of NAFLD and for the management of obesity-associated alterations.

## Figures and Tables

**Table 1:** *m*/*z* [M − H]^−^ ion, MS^n^ fragments of the compounds obtained by FIA-ESI-IT-MS/MS of the AERM.

*m*/*z* [M − H]^−^	MS²	MS³	Proposed Name
289	137 [M-152-H]^−^		catechin
435	283 [M-152-H]^−^	137 [M-152-146-H]^−^	catechin + deoxyhexose
451	299 [M-152-H]^−^	137 [M-152-162-H]^−^	catechin + hexose
515	353 [M-162-H]^−^	191 [M-162-162-H]^−^	dicaffeoyl-quinic acid
577	451 [M-126-H]^−^		catechin dimer
	425 [M-152-H]^−^		
	289 [M-288-H]^−^		
609	463 [M-146-H]^−^		rutin
	301 [M-308-H]^−^		
723	571 [M-152-H]^−^	419 [M-152-H]^−^	catechin dimer + deoxyhexose
739	587 [M-152-H]^−^	569 [M-18-H]^−^	catechin dimer + hexose
		435 [M-152-H]^−^	
865	577 [M-288-H]^−^	451 [M-126-H]^−^	catechin trimer
		425 [M-152-H]^−^	
		289 [M-288-H]^−^	
1153	865 [M-288-H]^−^	847 [M-18-H]^−^	catechin tetramer
		739 [M-126-H]^−^	
		587 [M-278-H]^−^	
		577 [M-288-H]^−^	
		451 [M-414-H]^−^	

**Table 2:** Body weight and body composition of control mice, control mice treated with AERM 5 mg·kg^−1^ (Control5) or 50 mg·kg^−1^ (Control50), obese mice (HFD) and obese mice treated with AERM 5 mg·kg^−1^ (HFD5) or 50 mg·kg^−1^ (HFD50).

Parameters	Control	Control5	Control50	HFD	HFD5	HFD50
Body weight at 8th week (g)	42.2 ± 1.4	41.7 ± 1.2	43.0 ± 1.8	55.0 ± 1.2 *	52.0 ± 2.0	53.0 ± 1.2
Final body weight (g)	44.7 ± 1.8	45.2 ± 1.6	43.5 ± 1.3	61.2 ± 2.4 *	56.0 ± 2.7	55.2 ± 1.3
∆ Body weight (%)	5.8 ± 1.2	8.2 ± 1.5	1.6 ± 2.5	9.8 ± 2.6	6.2 ± 2.7	4.2 ± 2.2
Food intake (kcal/day)	14.3 ± 1.1	13.3 ± 0.4	12.0 ± 0.4 ^#^	25.5 ± 1.1	24.3 ± 0.9	23.4 ± 1.9
Epididimal fat (g)	1.8 ± 0.3	1.5 ± 0.2	1.5 ± 0.1	2.7 ± 0.1 *	2.9 ± 0.2	2.7 ± 0.4
Epididimal fat (%) ^a^	4.0 ± 0.4	3.4 ± 0.3	3.7 ± 0.1	4.4 ± 0.1	5.1 ± 0.4	5.1 ± 0.8
Subcutaneous fat (g)	0.7 ± 0.1	0.5 ± 0.1	0.6 ± 0.1	1.3 ± 0.1 *	1.0 ± 0.1	1.2 ± 0.2
Subcutaneous fat (%) ^a^	1.6 ± 0.2	1.2 ± 0.1	1.5 ± 0.1	2.1 ± 0.1 *	1.8 ± 0.3	2.2 ± 0.2
Liver (g)	1.8 ± 0.1	1.9 ± 0.1	1.7 ± 0.1	3.0 ± 0.3 *	2.1 ± 0.1 ^#^	2.2 ± 0.2 ^#^
Liver (%) ^a^	4.2 ± 0.2	4.2 ± 0.1	4.0 ± 0.1	4.9 ± 0.3	3.7 ± 0.3 ^#^	3.9 ± 0.1 ^#^
Gastrocnemius muscle (g)	0.2 ± 0.0	0.2 ± 0.0	0.2 ± 0.0	0.2 ± 0.0	0.2 ± 0.0	0.2 ± 0.0
Gastrocnemius muscle (%) ^a^	0.5 ± 0.1	0.4 ± 0.0	0.5 ± 0.0	0.3 ± 0.0	0.4 ± 0.0	0.3 ± 0.0

^a^ % of body weight. The results are expressed as the means ± standard errors of the mean (*n* = 5). * *p* < 0.05 when compared with control group and ^#^ *p* < 0.05 when compared with non-treated group.

**Table 3:** Serum parameters and kITT of control mice, control mice treated with AERM 5 mg·kg^−1^ (Control5) or 50 mg·kg^−1^ (Control50), obese mice (HFD) and obese mice treated with AERM 5 mg·kg^−1^ (HFD5) or 50 mg·kg^−1^ (HFD50).

Parameters	Control	Control5	Control50	HFD	HFD5	HFD50
Fasting glucose (mg/dL)	166 ± 5	150 ± 4	152 ± 5	226 ± 11 *	201 ± 22	197 ± 6 ^#^
Fasting insulin (ng/mL)	106 ± 13	112 ± 14	207 ± 16 ^#^	176 ± 34 *	161 ± 17	127 ± 9 ^#^
kITT	5.4 ± 0.3	5.7 ± 0.7	5.0 ± 0.5	2.0 ± 0.4 *	2.6 ± 0.8	3.2 ± 0.3 ^#^
Total cholesterol (mg/dL)	174 ± 2	164 ± 2 ^#^	168 ± 2	203 ± 9 *	184 ± 7	180 ± 4 ^#^
LDL-cholesterol (mg/dL)	100 ± 7	98 ± 6	102 ± 6	144 ± 10 *	118 ± 4 ^#^	106 ± 9 ^#^
HDL-cholesterol (mg/dL)	57 ± 4	54 ± 3	52 ± 1	52 ± 2	50 ± 1	54 ± 2
Triglycerides (mg/dL)	187 ± 22	193 ± 32	201 ± 24	99 ± 7 *	98 ± 5	127 ± 17

The results are expressed as the means ± standard errors of the mean (*n* = 5). * *p* < 0.05 when compared with control group and ^#^ *p* < 0.05 when compared with non-treated group.

**Table 4:** Primers used for real-time PCR.

Gene	Primer	Sequence (5′ → 3′)
*Pparg*	Sense	GATGGAAGACCACTCGCATT
	Antisense	AACCATTGGGTCAGCTCTTG
*Ppara*	Sense	AGAAGTTGCAGGAGGGGATT
	Antisense	TTGAAGCAGCTTYGGGAAGA
*Srebf1*	Sense	GTGAGCCTGACAAGCAATCA
	Antisense	GGTGCCTACAGAGCAAGAGG
*Cd36*	Sense	ATTCTCATGCCAGTCGGAGA
	Antisense	TGGCTTTTGCACATCAAAGA
*Tnf*	Sense	TAGCCAGGAGGGAGAACAGA
	Antisense	TTTTCTGGAGGGAGATGTGG
*Ccl2*	Sense	CCCAATGAGTAGGCTGGAGA
	Antisense	TCTGGACCCATTCCTTCTTG
*Lep*	Sense	CTATGCCACCTTGGTCACCT
	Antisense	ACCAAACCAAGCATTTTTGC
*Il10*	Sense	ATCGATTTCTCCCCTGTGAA
	Antisense	TTCATGGCCTTGTAGACACCT
*Il6*	Sense	TCTCTGGGAAATCGTGGAA
	Antisense	TTCTGCAAGTGCATCATCG
*Fasn*	Sense	CACAGATGATGACAGGAGATGGA
	Antisense	TCGGAGTGAGGCTGGGTTGATA
*18s*	Sense	AAACGGCTACCACATCCAAG
	Antisense	CAATTACAGGGCCTCGAAAG

## Abbreviations

ABTS	2,2′-azino-bis(3-ethylbenzothiazoline-6-sulphonic acid)
AERM	Acetonic extract of *Rhizophora mangle* barks
DPPH	2,2-diphenyl-1-picrylhydrazyl
FA	Formic acid
FFA	Free-fat-acid
HFD	High-fat diet
HPLC-PDA	High-performance liquid chromatography coupled to photodiode array
HRF	Heterocyclic ring fission
FIA-ESI-IT-MS	Flow injection analysis electrospray-ion trap mass spectrometry
kiTT	Insulin tolerance test
*m*/*z*	mass/charge ratio
NAFLD	Non-alcoholic fatty liver disease
Pas	Proanthocyanidins
PCR	Polymerase chain reaction
RDA	Retro Diels-Alder
ROS	Reactive oxygen species
SPE	Solid-phase extraction
T2D	Type 2 diabetes
TEAC	Trolox equivalent antioxidant activity
QM	Quinone-methide
