# Addendum: de Souza Mesquita, L.M., et al. Modulatory Effect of Polyphenolic Compounds from the Mangrove Tree *Rhizophora mangle* L. on Non-Alcoholic Fatty Liver Disease and Insulin Resistance in High-Fat Diet Obese Mice. *Molecules*, 2018, *23*, 2114

**DOI:** 10.3390/molecules24010169

**Published:** 2019-01-04

**Authors:** Leonardo Mendes de Souza Mesquita, Cíntia Rabelo e Paiva Caria, Paola Souza Santos, Caio Cesar Ruy, Natalia da Silva Lima, Débora Kono Taketa Moreira, Claudia Quintino da Rocha, Daniella Carisa Murador, Veridiana Vera de Rosso, Alessandra Gambero, Wagner Vilegas

**Affiliations:** 1 UNESP—São Paulo State University/Coastal Campus of São Vicente, Laboratory of Bioprospection of Natural Products (LBPN) Pça Infante Dom Henrique S/N, 11330-900 São Vicente, SP, Brazil; mesquitalms@gmail.com (L.M.d.S.M.); claudiarocha3@yahoo.com.br (C.Q.d.R.); 2 Clinical Pharmacology and Gastroenterology Unit, USF—São Francisco University, Av. São Francisco de Assis, 218, 12916-900 Bragança Paulista, SP, Brazil; cintiarabello@yahoo.com.br (C.R.e.P.C.); pa.s.santos@hotmail.com (P.S.S.); ruyccaio@gmail.com (C.C.R.); lima.nat@gmail.com (N.d.S.L.); deboraktmoreira@gmail.com (D.K.T.M.); alessandra.gambero@usf.edu.br (A.G.); 3 Laboratório de Estudos Avançados em Fitomedicamentos (LEAF), UFMA—Federal University of Maranhão, Av. dos Portugueses, 1966-Bacanga, CEP: 65080-805 São Luís, Maranhão, Brazil; 4 Department of Biosciences, Federal University of São Paulo (UNIFESP), Silva Jardim Street, 136, Vila Mathias, 11015-020 Santos City, SP, Brazil; daniellamurador@gmail.com (D.C.M.); veriderosso@yahoo.com (V.V.d.R.)

The authors wish to add the process number in the Acknowledgments section in this paper [1]. The corrected acknowledgement is provided below:

**Acknowledgments:** This work was supported by “Fundação de Amparo a Pesquisa do Estado de São Paulo—FAPESP for funding (2009/52237-9), (2018/16388-1) and fellowships (2014/23951-3). 

The authors would like to apologize for any inconvenience caused to the readers by these changes.
